# Enhancing the Activity of Silver Nanowire Membranes by Electrochemical Cyclic Voltammetry as Highly Sensitive Flexible SERS Substrate for On-Site Analysis

**DOI:** 10.3390/nano11030672

**Published:** 2021-03-09

**Authors:** Rui Zhang, Yongchao Lai, Jinhua Zhan

**Affiliations:** 1 Key Laboratory for Colloid & Interface Chemistry of Education Ministry, Department of Chemistry, Shandong University, Jinan 250100, China; zr62956@163.com; 2 Shandong Academy of Occupational Health and Occupational Medicine, Shandong First Medical University & Shandong Academy of Medical Sciences, Jinan 250062, China

**Keywords:** surface-enhanced Raman spectroscopy, nanostructures reconstitution, silver nanowires, cyclic voltammetry, swabbing extraction

## Abstract

The development of high-quality flexible surface-enhanced Raman spectroscopy (SERS) substrates is crucial for developing rapid SERS analysis in situ. Silver nanowire membranes as novel flexible substrates could benefit from the high collection efficiency of analytes by wrapping complex surfaces or wiping the surfaces of samples. However, their low SERS performance impedes further applications of silver nanowire membranes in analyte detection. Herein, we report an ultra-high-sensitivity silver nanowire membrane synthesized by a simple and time-saving cyclic voltammetry (CV) method. After CV treatment, a part of the silver nanowires on the silver nanowire membrane turned into small nanoparticles and nanorods. This nanostructure’s reconstitution increased the analytical enhancement factor of silver nanowire membranes by 14.4 times. Scanning and transmission electron microscopy, UV-vis spectroscopy, X-ray diffraction, and X-ray photoelectron spectroscopy were employed to investigate the transformation in the membrane nanostructure. The CV-treated substrates exhibited high surface-enhanced Raman activity and good temporal stability. The limits of detection (LODs) for *p*-aminothiophenol, crystal violet, tetramethylthiuram disulfide, sodium perchlorate, malachite green, fluoranthene, and potassium nitrate are 3.7 × 10^−12^ M, 5.1 × 10^−11^ M, 5.4 × 10^−11^ M, 6.3 × 10^−9^ M, 0.00693 ng, 0.0810 ng, and 0.0273 ng on this substrate, respectively. Additionally, the developed substrate is feasible for the detection of crystal violet in real samples. These results certify that CV-treated substrates possess broad application prospects in on-site SERS analysis.

## 1. Introduction

Surface-enhanced Raman spectroscopy (SERS) has been widely used to detect trace biological and chemical compounds since it was discovered. Nowadays, SERS plays an increasingly important role in various fields such as life sciences, environmental monitoring, and medical diagnosis [1,2,3]. The cornerstone of SERS detection is the fabrication of high-quality SERS substrates. Generally, gold or silver plasmonic nanostructures are used to fabricate SERS substrates because they can generate high local electromagnetic fields, owing to the localized surface plasmon resonance (LSPR) effect. Thus, these substrates enhance the Raman signals of molecules on the surface of substrate through electromagnetic enhancement mechanisms [4]. The activity of a SERS substrate is strongly correlated with the morphology of its constituent plasmonic nanostructures [5,6,7,8,9,10]. A variety of SERS substrates have been customized to suit various SERS applications. In particular, flexible substrates have attracted special attention for the practical application of SERS because they can be deformed to better fit the surfaces of interest and collect hazardous materials effectively by wrapping or wiping during on-site detections [11,12,13,14,15,16,17,18]. Moreover, these flexible substrates can be easily cut into specimens of different sizes and shapes as required and can be easily integrated with other devices [19,20,21]. Common flexible SERS substrates mainly include plasmonic nanostructures and flexible substrates, such as polymer films, carbon nanotubes, graphene, cellulose, and filter paper [22].

As novel flexible substrates, membranes of silver nanowires have attracted increasing attention because silver nanowires with a certain aspect ratio can be easily loaded onto a filter membrane to generate a flexible SERS substrate without complex fabrication processes [23,24,25,26,27,28]. The assembled silver nanowire membranes have a three-dimensional multilayer configuration and produce a strong electromagnetic field enhancement effect (SERS hot spots) at the cross points of silver nanowires [29]. Therefore, SERS hot spots exist in a minority of regions of the silver nanowire membrane, and there is still a big potential to obtain a higher density of hotspots in the membrane. Although silver nanowire membranes have been used in many fields for chemical analysis, the low density of SERS hot spots still impedes their widespread applications. There have been numerous research studies to increase the density of SERS hot spots on silver nanowires, including the fabrication of gold nanoparticle/silver nanowire heterostructures, and the conversion of disordered silver nanowires into ordered ones at the oil–water–air interface [29,30]. However, these processes are tedious and time-consuming, and they hinder further applications of silver nanowire membranes.

Electrochemical cyclic voltammetry (CV), a simple and commonly used method, can reform the plasmonic nanostructures of SERS substrates through electrocorrosion and electroplating processes to improve the sensitivity of SERS substrates [31,32]. Herein, electrochemical CV is used to treat silver nanowire membranes to transform a part of the silver nanowires into silver nanorods and nanoparticles. The treated substrates show strong surface plasmon resonance absorption and high surface-enhanced Raman activity, revealing that the electrochemical treatment can significantly increase the density of hot spots of such substrates. The analytical enhancement factor (AEF) of the substrate reaches a value of 1.24 × 10^9^ on average, which is 14.4 times higher than that of the untreated substrate. In this study, the limits of detection (LODs) for p-aminothiophenol, crystal violet, tetramethylthiuram disulfide, sodium perchlorate, malachite green, fluoranthene, and potassium nitrate are 3.7 × 10^−12^ M, 5.1 × 10^−11^ M, 5.4 × 10^−11^ M, 6.3 × 10^−9^ M, 0.00693 ng, 0.0810 ng, and 0.0273 ng on this substrate, respectively. Moreover, the feasibility of the developed substrate for crystal violet detection in real samples is also demonstrated. This highly sensitive and flexible SERS substrate may find more applications in the on-site detection of analytes in the future.

## 2. Materials and Methods

### 2.1. Chemicals and Materials

Silver nitrate (99.8%), polyvinylpyrrolidone K-30 (PVP, Mw = 55000, GR), glycerol (99%), sodium chloride (99.8%), sodium diethyldithiocarbamatre (DDTC, AR) and sodium borohydride (AR) were purchased from Sinopharm Chemical Reagent Co. Ltd. (Shanghai, China). Ethanol (99.7%) was purchased from Tianjin Fuyu Fine Chemical Co., Ltd. (Tianjin, China). *p*-Aminothiophenol (PATP, 98%), crystal violet (99%), sodium perchlorate (AR), tetramethylthiuram disulfide (thiram, 99%), malachite green (AR), potassium nitrate (AR), fluoranthene (AR), 1-propanethiol (99.5%), and methanol (99.9%) were obtained from Aladdin Chemicals (Shanghai, China). Ag/AgCl reference electrode and electrode clamp (Platinum plate, PTFE) were purchased from Wuhan GaossUnion Technology Co., Ltd. (Wuhan, China) Titanium sheet (99.99%) was obtained from Jinbu Titanium Nickel Manufacturing Co., Ltd. (Baoji, China). The quantitative filter paper (Model: 203) was purchased from Shanghai Leigu Instrument Co., Ltd. (Shanghai, China). Ultrapure water (18.25 MΩ∙cm^−1^) was used in all steps.

### 2.2. Instrumentation

The morphology of the silver nanowires was characterized using a field-emission scanning electron microscope (JSM-6700F, Japan Electronics Corporation, Tokyo, Japan) equipped with an energy-dispersive spectroscope (Oxford INCA X sight instrument, Oxford Instruments GmbH, Wiesbaden, Germany). The X-ray diffraction (XRD) patterns were recorded on a Bruker D8 advanced X-ray diffractometer (Bruker AXS, Karlsruhe, Germany) equipped with graphite monochromatized Cu-*K*_α_ radiation (λ = 1.5418 Å). XPS was performed on a Thermo Fisher scientific ESCALAB 250 spectrophotometer (Thermo Fisher Scientific Inc., Waltham, MA, USA). The UV-vis and diffuse reflection spectroscopy was conducted on a Hitachi U-4100 (Hitachi, Ltd., Tokyo, Japan). All Raman measurements were performed with an Ocean Optics QE65000 spectrometer (Ocean Optics, Dunedin, FL, USA), except for the temporal stability test. The excitation wavelength was 785 nm and the input laser source of the instrument was operated at 50 mW. The diameter of the focused laser beam was about 158 μm. A 7.5 mm objective lens was used, and the integration time was 1 s for recording the SERS spectra. Electrochemical experiments were performed on a Princeton PARSTAT3000A electrochemical workstation (AMETEK Scientific Instruments, Princeton, NJ, USA). The temporal stability was examined using a DXR2 confocal Raman microscope (Thermo Fisher Scientific Inc., Waltham, MA, USA) with a 785 nm laser and a 10× objective lens (NA/0.25). The excitation power is 10 mW and the acquisition time of each spectrum was 1 s. The interval of data recording was 5 s.

### 2.3. Preparation of Silver Nanowires

Silver nanowires were synthesized by a water-modulated solvothermal polyol process [33]. Typically, 3.52 g of PVP-K30 was added to 116 mL glycerol in a 250 mL round-bottom flask and stirred gently at 85 °C until the solid dissolved completely. Then, silver nitrate (0.868 g) was added to the solution after cooling it to room temperature. Subsequently, a glycerol solution (4 mL) containing 35.4 mg of sodium chloride and 0.15 mL of water was added to the solution. Then, the flask was immersed in an oil bath and stirred with a polytetrafluoroethylene magneton. Then, the solution was heated to 210 °C for 20 min. Once the temperature of the solution reached 210 °C, the solution was allowed to cool spontaneously to room temperature. Then, 120 mL of water was added to the flask with gentle stirring, and the product was allowed to precipitate over 48 h. Thereafter, the precipitate was collected and washed thrice with water. The as-obtained silver nanowires were finally dispersed in 100 mL of ethanol.

### 2.4. Preparation of CV Treated Silver Nanowire Membranes

A silver nanowire membrane was developed by filtering 2 mL of ethanolic dispersion of the silver nanowires through a quantitative filter paper (diameter = 25 mm, maximum aperture diameter: 10–15 µm). Next, the membrane was immersed in 1% (mass fraction) newly prepared sodium borohydride solution for 5 min and dried naturally to remove the PVP ligands [34]. Then, the membrane was cut into 10 mm × 5 mm rectangles and used as working electrodes. An electrolytic cell with three electrodes was used for treating the membrane and recording the current–voltage curves. An Ag/AgCl electrode was used as the reference electrode; a 100 mL beaker filled with 100 mL of 0.1 mol·L^−1^ hydrogen chloride solution was used as the electrolytic cell, and a titanium sheet covering the inner curved surface of the beaker was used as the auxiliary electrode. Then, the cut membrane was fixed on the platinum electrode holder to maintain good contact between the silver nanowires membrane and platinum sheet. Additionally, the area immersed in the electrolyte is 5 mm × 5 mm. During the experiment of treating the membrane in different scanning cycles at constant temperature, the experiment method was cyclic voltammetry; the potential scan rate was 0.025 V∙s^−1^; the potential scan range was from −0.1323 to 0.2677 V; the number of scans was set at 5, 10, 15, 20, and 25, respectively; the beaker was immersed in a water bath at 20 °C. During the experiment of treating the membrane in constant different scanning cycles at different constant temperature, the experiment method, the potential scan rate and the potential scan range were not changed; the number of scans was set at 15; the water bath was set at 16, 18, 20, 22, and 24 °C, respectively. Every time before starting the CV scan, the temperature of the beaker was ensured to reach the preset value and kept constant for 5 min. After the treatment, the treated substrates were washed with ultrapure water for 3 times and then dried naturally.

### 2.5. SERS Performance of CV Treated Silver Nanowire Membranes

The SERS detections of *p*-aminothiophenol, crystal violet, tetramethylthiuram disulfide, and sodium perchlorate were achieved by immersing the substrate in a large volume of aqueous solution with specific concentrations for 2 h. Then, these substrates were dried in air for detection. The signals of *p*-aminothiophenol, crystal violet, and tetramethylthiuram disulfide were collected and used directly without normalization. Substrates were decorated with DDTC by immersing them in a 10 mM aqueous DDTC solution for 2 h before detecting sodium perchlorate, and the SERS signals were normalized by Raman peak at 1271 cm^−1^ of DDTC [12].

The swabbing extraction SERS detection of malachite green, fluoranthene [35], and potassium nitrate [12] were achieved by swabbing them on clean glass, polyethylene packaging bag, and aluminum sheet, respectively. The substrates were wet with methanol when swabbing fluoranthene and infiltrated with water while swabbing malachite green and potassium nitrate. The signals of malachite green were collected and used directly without normalization. Briefly, for the malachite green detection, 10 µL of 10^−8^–10^−3^ M of malachite green aqueous solution were dropped on a clean glass and left to dry naturally. Then, the optimized substrate soak with water was used to gently swab the glass surface to collect the residue of the analytes. One gentle swabbing is sufficient. Substrates were decorated with propyl mercaptan by immersing them in a 10 mM ethanolic propyl mercaptan solution for 2 h before detecting fluoranthene and the SERS signals obtained were normalized by Raman peak at 1025 cm^−1^ of propyl mercaptan. Briefly, 10 µL of 10^−7^–10^−3^ M of fluoranthene ethanolic solution were dropped on a polyethylene packaging bag and left to dry naturally. Then, the optimized decorated substrate soak with ethanol was used to gently swab the bag surface to collect the residue of the analytes. One gentle swabbing is sufficient. Before detecting potassium nitrate, the substrate must be modified with DDTC by the infiltration in a 10 mM DDTC aqueous solution for 2 h. Then, the SERS signals obtained were normalized by Raman peak at 1271 cm^−1^ of DDTC. Briefly, 10 µL of 10^−8^–10^−3^ M of potassium nitrate aqueous solution were dropped on an aluminum sheet and left to dry naturally. Then, the optimized decorated substrate soak with water was used to gently swab the sheet surface to collect the residue of the analytes. One gentle swabbing is sufficient. During all the SERS detections, the excitation wavelength was 785 nm and the input laser source of the instrument was operated at 50 mW, and the integration time was 1 s.

### 2.6. Simulations

The simulations were carried by DDSCAT 7.3 [36]. It was written in Fortran by Draine and Flatau from Princeton University and runs on Linux. DDSCAT 7.3 is a freely available Fortran-90 open-source software that uses the discrete dipole approximation (DDA) to calculate the electromagnetic wave scattering and absorption of targets with arbitrary geometric shapes and complex refractive index. The complex permittivity values in simulations are referred from Palik [37].

## 3. Results and Discussion

### 3.1. Characterization of CV-Treated Silver Nanowire Membrane

**Figure 1:**
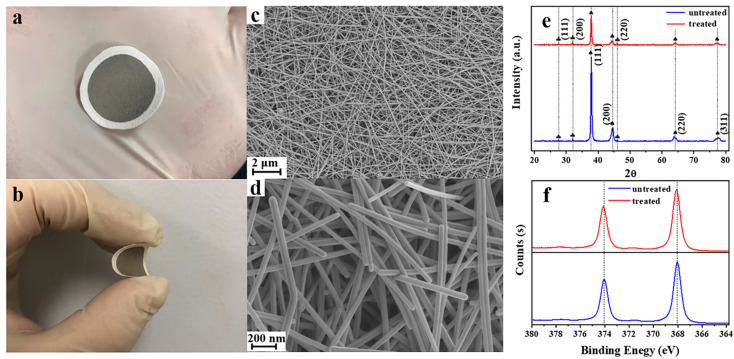
Optical photos of flexible silver nanowire membrane (**a**,**b**). (**c**) Low- and (**d**) high-magnification SEM images of a silver nanowire membrane. XRD patterns of the silver nanowire membrane before and after cyclic voltammetry (CV) treatment (cycle number: 15; temperature: 20 °C) (**e**) and the XPS of the silver nanowire membrane before and after CV treatment (cycle number: 15; temperature: 20 °C) (**f**).

Scanning electron microscopy (SEM), transmission electron microscopy (TEM), and energy-dispersive X-ray spectrometry (EDS) were employed to characterize the structure and morphology of silver nanowires and their membranes. The SEM images of the untreated silver nanowire membrane at different magnifications are shown in Figure 1c,d. As-prepared silver nanowires were observed to have a smooth surface, a length of few microns to tens of microns, and a relatively uniform diameter of ≈60 nm, which was also confirmed by the TEM images (Figure S1). The membranes based on filter paper packed randomly with silver nanowires have a dense structure and tiny pores.

The Uv-vis absorption spectrum of the silver nanowire aqueous colloidal dispersion is shown in Figure S2. There are two peaks at 354 nm and 384 nm.

The XRD patterns of the silver nanowire membrane before and after subjecting to CV are shown in Figure 1e. The peaks marked with spades in the patterns are attributed to silver-3C (JCPDS No. 87-0597). The comparatively weak characteristic peaks marked with clubs, which appear both in the spectra of untreated and treated silver nanowire membranes, could be assigned to chlorargyrite (JCPDS No. 85-1355). These results are consistent with previous reports wherein silver chloride species are formed at the very beginning of the synthesis and serve as crystal nuclei for the growth of silver nanowires. After CV treatment, silver chloride still exists on the surface of the silver nanowire membrane. It can also be observed that the peaks of silver-3C weaken after CV treatment, indicating that the crystallinity of the silver nanowire membrane decreases after CV treatment. The decrease in the crystallinity of silver nanowires can be attributed to the transformation of some silver nanowires into silver nanoparticles and nanorods.

The X-ray photoelectron spectra (XPS) of the silver nanowire membranes before and after CV treatment are shown in Figure 1f. The peak positions of silver did not change significantly (≤0.05 eV) after CV treatment, indicating that the chemical state of silver on the substrate did not change after the CV treatment.

The EDS of the silver nanowire membrane treated by CV with 15 cycles at 20 °C is shown in Figure S3. It can be learnt from the figure that the element Ag and Cl are distributed evenly on the substrate surface. The content of Ag is 97.7% and that of Cl is 2.3%.

### 3.2. Effect of Electrochemical Conditions on the Silver Nanowires Membrane

Electrochemical treatment of the silver nanowire membrane was carried out by CV. CV is often used to reform precious metal materials to increase the performance of SERS substrates [38,39,40]. In this method, the scanning voltage within a certain potential range is applied repeatedly when the silver nanowire membrane used as the working electrode in a three-electrode system alternately undergoes oxidation and reduction reactions multiple times. As shown in Figure S4, the current–potential curve, current–time curve, charge–time curve, and potential–time curve were recorded at the potential scan rate of 0.025 V∙s^−1^; the potential scan range was from −0.1323 to 0.2677 V, the number of scans was 15, and the temperature at which the measurement was carried out was 20 °C. The substrate, which is the silver nanowire membrane, underwent cyclic oxidation and reduction during the applied potential range. The main reactions occurring during this potential scan are depicted in Equations (1) and (2).

Oxidation step: Ag + Cl^−^ − e^−^ → AgCl (1)

Reduction steps: AgCl + e^−^ → Ag + Cl^−^ (2)

As the reaction system contains a low concentration of chloride ions, elemental silver is oxidized to silver chloride in the oxidation step (Equation (1)), and silver chloride is reduced to elemental silver in the reduction step (Equation (2)). Thus, the plasmonic nanostructures on the silver nanowire membrane can be refactored by the CV process. It should be noted that the current and charge decreased gradually with each cycle, which suggests that the overall resistance of the membrane increased slowly, which might be due to the conversion of silver nanowires into silver nanoparticles and nanorods.

**Figure 2:**
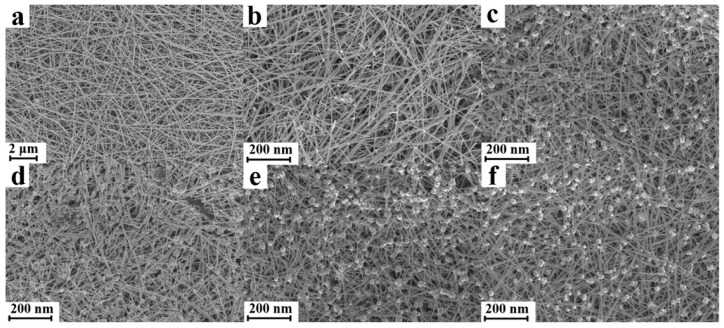
SEM images of silver nanowire membranes according to the CV cycle number: untreated (**a**), 5 cycles (**b**), 10 cycles (**c**), 15 cycles (**d**), 20 cycles (**e**), and 25 cycles (**f**).

The SEM images of the silver nanowire membrane subjected to different CV cycles (20 °C) are shown in Figure 2. It is clear that with an increase in the cycle number, the silver nanowires turned into small nanoparticles and nanorods. Nanoparticles and nanorods appeared on the substrate when the cycle number was 5, and the number of nanoparticles and nanorods increased up to the cycle number of 15. However, with a further increase in the cycle number, the number of nanoparticles and nanorods ceased to increase, and the sizes of nanoparticles and nanorods increased instead. In the first 15 cycles, the silver atoms being electrolyzed mainly originated from silver nanowires in the oxidation step, and the released silver ions recrystallized to form silver nanoparticles and nanorods. With a further increase in the number of CV cycles, the silver ions first deposited on the surface of the pre-existing silver nanoparticles and silver nanorods owing to the high nucleation energy barrier for the formation of new nanoparticles. It should be noted that appropriately sized silver nanoparticles and silver nanorods will produce a strong SPR in the visible region, especially when they form nanoscale gaps among one another. As abundant silver nanoparticles and nanorods partially substitute the silver nanowires on the surface of the membrane, a significant increase in the density of hotspots and the SERS performance of the silver nanowire membrane are expected.

Further, silver nanowire membranes treated at different temperatures (15 cycles) were also investigated, and the corresponding SEM images are illustrated in Figure S5. Different surface morphologies were observed when the temperature was changed. When treated at high temperatures (>20 °C), the silver nanoparticles and nanorods tended to aggregate and grow into larger particles (Figure S5a–c). Meanwhile, a smaller number of nanoparticles and nanorods were produced at low temperatures (<20 °C, Figure S5d–e). Neither of these two situations led to the creation of many hotspots to maximize the SERS performance of silver nanowire membrane.

**Figure 3:**
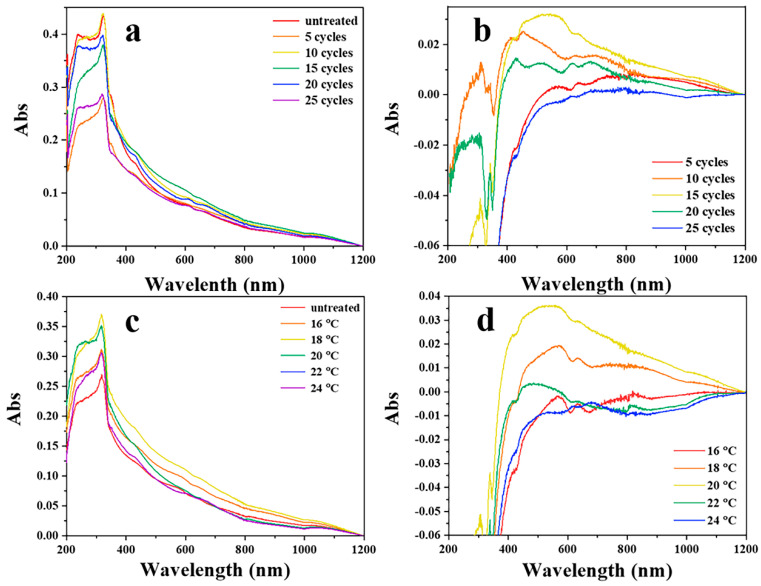
The diffuse reflection spectroscopy of silver nanowires membranes treated with different cycle numbers (**a**) and at different temperatures (**c**). Absorption spectrum by subtracting the absorption spectrum of the untreated substrate from that treated with different cycles (5, 10, 15, 20, and 25) by using cyclic voltammetry (**b**). Absorption spectrum by subtracting the absorption spectrum of the untreated substrate from that treated at different temperatures (16, 18, 20, 24, and 26 °C) by using cyclic voltammetry (**d**).

As the SPR depends strongly on the shape and size of the plasmonic nanostructure, diffuse reflection spectroscopy was performed to analyze the SPR of substrates and to characterize the change in the surface nanostructures after CV treatment. The diffuse reflection spectra of the silver nanowire membranes are shown in Figure 3a (after different CV cycles) and Figure 3c (CV performed at different temperatures). Differences in the absorption between the untreated and treated substrates basically represent the light absorption by the newly generated nanostructures after CV treatment. Substrates prepared under different conditions may have different surface morphologies. In order to achieve a sharper contrast between the spectra of treated and untreated samples, a series of spectra were obtained by subtracting the absorption spectra of the treated substrates with that of the untreated one. As shown in Figure 3b,d, the absorption band between 400 and 500 nm may be attributed to the characteristic absorption of silver nanoparticles. The absorption band ranging from 600 to 1000 nm might arise from the long-axis absorption of silver nanorods. Figure 3 indicates that the substrate has a comparatively high SPR absorbance between 400 and 500 nm when the CV cycle number is 15 and the temperature is 20 °C. Combined with the results of SEM images shown before (Figure 2d and Figure S5c), the optimum conditions for treating the substrates were deemed to be 15 CV cycles at 20 °C.

### 3.3. SERS Performance of the Electrochemically Treated Silver Nanowire Membrane

Before carrying out the experiments about SERS performance, a detailed SERS spectrum of the as-prepared substrate should be collected. Figure S6 shows the SERS spectra of raw substrate (red) and substrate washed by sodium borohydride (blue). The black line is background. It can be seen that the interference of impurities is greatly reduced after adding sodium borohydride. All the substrates used in the next experiment were washed by sodium borohydride [34].

**Figure 4:**
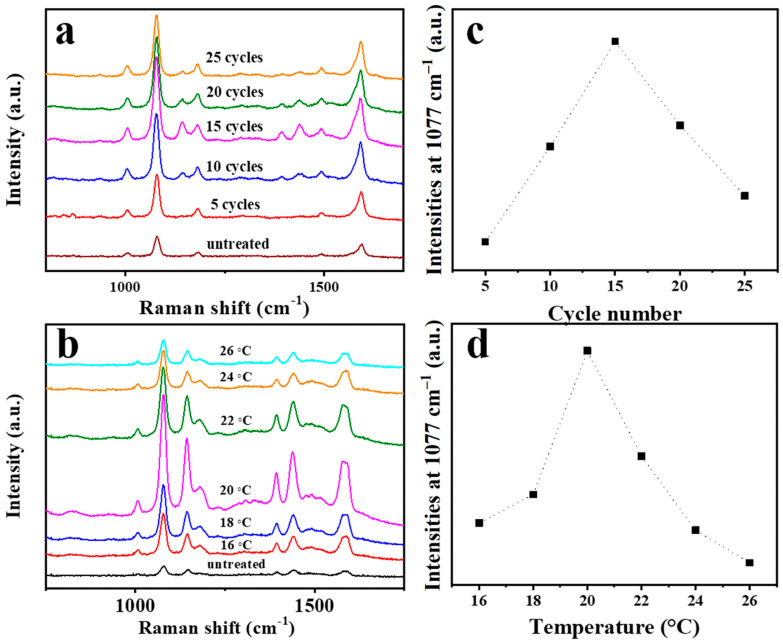
Surface-enhanced Raman spectroscopy (SERS) spectra of p-aminothiophenol (10^−6^ M) used as a probe molecule on silver nanowire membranes according to the CV cycle number (**a**) and treatment temperature (**b**). SERS intensities at 1077 cm^−1^ as a function of the cycle number (**c**) and treatment temperature (**d**).

*p*-Aminothiophenol (PATP) was chosen as the probe molecule to evaluate the SERS activity of the silver nanowire substrates subjected to different scanning cycles (5, 10, 15, 20, and 25 CV cycles) at a scan rate of 0.025 V∙s^−1^ at 20 °C. As shown in Figure 4a, the SERS signal of PATP increases gradually as the CV scan cycle increases up to the 15th cycle and then decreases. In addition, the temperature of the electrolyte during the CV experiment was also found to affect the SERS performance of the prepared substrate. In order to investigate the relationship between the temperature and Raman activity, substrates treated by CV at different temperatures (16, 18, 20, 22, 24, and 26 °C) were immersed in 10^−6^ M PATP for 2 h. The SERS spectrum of each substrate is shown in Figure 4b. The SERS performance of the substrate increases at first and then decreases with a further increase in the temperature, with the optimal performance being achieved at the temperature of 20 °C. Therefore, 15 CV cycles at 20 °C were chosen as the optimal conditions for treating the silver nanowire membrane. Figure 4c,d show the SERS intensities at 1077 cm^−1^ as a function of the cycle number and treatment temperature.

The AEF that was used to evaluate the SERS performance of the substrate is defined as follows [41]:

(3) $$AEF=\frac{I_{SERS}}{I_{Raman}}\times \frac{C_{Raman}}{C_{SERS}}.$$

Here, $I_{SERS}$ is the SERS intensity of 10^−6^ M PATP on the substrate; $I_{Raman}$ is the Raman intensity of PATP; $C_{Raman}$ is the concentration of PATP that participates in the normal Raman process; $C_{SERS}$ is the concentration of PATP that participates in the SERS process. The AEF values of the substrates prepared at different temperatures were evaluated from the intensity of the characteristic peak of PATP at 1076 cm^−1^ on each type of substrate. As shown in Tables S1–S3, CV treatment significantly increased the enhancement factor of the substrates; the maximum AEF is 1.24 × 10^9^, which is 14.4 times higher than that of the untreated substrate (8.6 × 10^7^).

**Figure 5:**
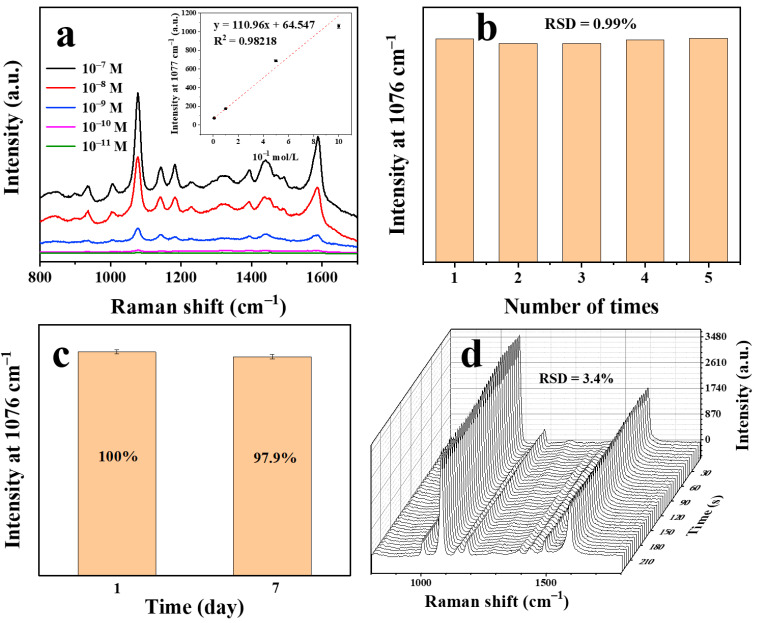
SERS spectra of *p*-aminothiophenol on silver nanowire membranes treated by 15 CV cycles at 20 °C (**a**) (Inset: the calibration curve of the *p*-aminothiophenol (PATP) detection). Reproducibility of the CV-treated silver nanowire membranes (**b**). Stability of the CV-treated silver nanowire membranes in air (**c**). Stability of the CV-treated silver nanowire membranes under laser (**d**).

To evaluate the SERS activity of the silver nanowire membrane treated under the optimal CV conditions, the substrates were exposed to different concentrations of PATP (10^−7^–10^−11^ M); The corresponding SERS spectra are shown in Figure 5a. The positions of the peaks are basically consistent with those reported in the literature [42]. As is shown in the insect of Figure 5a, taking SERS intensities at 1077 cm^−1^ into consideration, the SERS signal intensities linearly decrease with the increasing of negative logarithm of PATP concentration within this concentration range. The linear equation was given in the inset of Figure 5a within the range of 10^−11^–10^−9^ M and the limit of detection (LOD) of the PATP concentration was discovered to be 3.7167 × 10^−12^ M (LOD = 3*δ*/*S*, where *δ* is the blank standard deviation and *S* is the slope of the calibration curve) [43]. The conventional Raman spectrum of PATP is displayed in Figure S10a for comparison.

In order to confirm the reproducibility of the SERS sensor, PATP was detected in five replicate samples with a standard sample concentration of 10^−6^ M. As shown in Figure 5b, the SERS intensity distribution at 1077 cm^−1^ shows a low relative standard deviation (RSD) of 0.99%, indicating that the SERS intensity change of the proposed SERS sensor is very small. The temporal stability of the SERS substrate was investigated by collecting the spectra of PATP on the SERS substrates exposed to air and under continuous laser irradiation. The SERS intensity stability of the substrate was investigated. The SERS intensities of 10^−6^ M PATP with fresh substrate and old (exposed to air for 7 days) substrate were recorded (Figure 5c). The substrate exhibited good time stability. Furthermore, as shown in Figure 5d, there is no significant change in the spectra within 240 s. Taking the peak intensity at 1077 cm^−1^ as the reference, the RSD was calculated to be 3.4%. Thus, the substrates showed high sensitivity and reliable stability, which could facilitate rapid SERS analysis with this substrate.

The homogeneity of the SERS substrate was investigated by sampling in five locations and intensity mapping. As shown in Figure S7, the RSD of sample points was calculated to be 4.0%, which conveys that the substrate exhibits a good homogeneity on a larger scale. It is shown in Figure S8 that the substrate is still relatively uniform on the micron scale.

### 3.4. SERS Detection of Simulated Aqueous Samples

**Figure 6:**
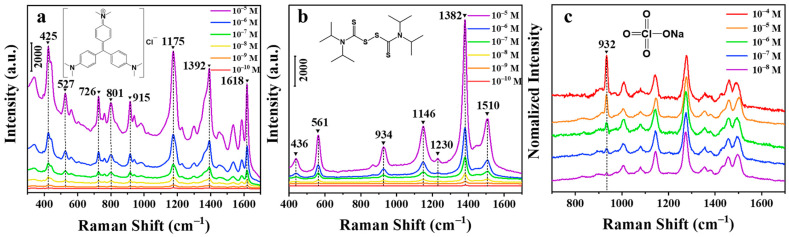
SERS spectra of crystal violet (**a**), thiram (**b**), and sodium perchlorate (**c**) on silver nanowire membranes treated by 15 CV cycles at 20 °C.

Crystal violet is an alkaline triphenylmethane disinfectant that has a significant bactericidal effect, especially against fungal infections and parasitic diseases in fish [44]. The results of recent studies show that crystal violet can be metabolized into fat-soluble stealth crystal violet through biotransformation after entering the fish body and has potential side effects such as carcinogenesis and mutagenesis [45]. Therefore, it is significant to detect the residue of crystal violet in cultured water. Since the crystal violet molecules are easily adsorbed on the silver surface, the substrates were immersed in 10^−5^ M to 10^−10^ M crystal violet aqueous solutions, and their corresponding SERS spectra were recorded. The Figure 6a shows the SERS spectrum of crystal violet with different concentrations on the surface of the silver nanowire membrane treated by CV and the illustration is of the molecular structure. The conventional Raman spectrum of crystal violet is exhibited in Figure S10b for comparison. The characteristic peaks of crystal violet are 425, 527, 726, 801, 915, 1175, 1392, and 1618 cm^−1^. Among them, the peak at 421 cm^−1^ is attributed to benzene–C–benzene out-of-plane deformation vibration; peaks at 527 and 915 cm^−1^ are owing to the aromatic ring skeleton vibration; the peaks at 726 and 1175 cm^−1^ originate from the radial plane aromatic ring carbon–hydrogen bond bending vibration; the peaks at 1618 cm^−1^ arise from C–C in-plane stretching vibration [46]. It can be seen from the Figure 6a that the characteristic peak intensity of crystal violet gradually increases with an increase in its concentration, and the linear equation was given in Figure S9a within the range of 10^−10^–10^−8^ M, and the LOD of the crystal violet concentration was found to be 5.1 × 10^−11^ M. Table S4 exhibits a comparison of performance of the developed SERS substrate with other sensors reported in literature for the detection of crystal violet.

Tetramethylthiuram disulfide (thiram) is a highly efficient and low-toxic pesticide, which is widely used in the prevention and control of crop diseases. However, the excessive residues of thiram in the crops will pose a serious threat to human health, and therefore, the detection of thiram is very important [47]. Figure 6b shows the SERS spectra of thiram in different concentrations on the electrochemically treated silver nanowire film. The illustration is of the molecular structure of thiram. The characteristic peaks of thiram are 439, 563, 928, 1149, 1229, 1392, and 1510 cm^−1^. Among them, the peak at 563 cm^−1^ is owing to the stretching vibration of the S-S bond; the peak at 928 cm^−1^ is attributed to the stretching vibration of CH_3_N; the peak at 1149 cm^−1^ arises from the stretching vibration of the C–N bond and CH_3_ plane; peaks at 1382 and 1510 cm^−1^ originate from the C–N stretching vibration and CH_3_ deformation vibration [48]. As is displayed in Figure 6b, the characteristic peak intensity of thiram increases with the increasing of its concentration. In addition, the linear equation was displayed in Figure S9b within the range of 10^−10^–10^−8^ M, and the LOD of the thiram concentration was calculated to be 5.4 × 10^−11^ M.

Some inorganic salt anions are relatively common pollutants in the environment. Among them, perchlorate is a typical toxic chemical substance. It is used extensively in rocket fuel and spreads into the environment upon fuel burning, which is harmful to humans and other species. Perchlorate can interfere with the synthesis and secretion of thyroxine, affect the body’s normal metabolism, and hinder growth and development [49]. Therefore, the detection of perchlorate is of great significance to human health. Figure 6c is the SERS spectra of different concentrations of sodium perchlorate (normalized by Raman peak at 1271 cm^−1^ of DDTC). Since perchlorate ions cannot be directly adsorbed on the surface of the substrate, DDTC was chosen as a modifier to enrich the perchlorate on the surface of the silver nanowire film by electrostatic action to achieve trace detection [50]. The inset is the structural formula of a perchlorate ion. It can be learnt from the figure that the characteristic peak intensity of perchlorate gradually is a proportional to its concentration. The linear equation was given in Figure S9c and the dynamic range was from 10^−9^ to 10^−7^ M. The LOD of the thiram concentration was discovered to be 6.3 × 10^−9^ M.

To further estimate the feasibility and applicability of the data obtained with the CV treated SERS substrate, pond water from nearby was spiked with various concentrations of crystal violet using the standard addition method. Combining the working curve of crystal violet obtained above (Figure S9a), the concentrations of samples were calculated. The relative standard deviation was calculated with five replicates of the test. As is shown in Table S5, recoveries range from 88.07% to 101.5%; RSDs range from 1.30% to 5.99%. The results demonstrated that the developed substrate is capable of testing trace crystal violet in real sample.

### 3.5. Swabbing Extraction-Based SERS Detection of Simulated Solid Samples

**Figure 7:**
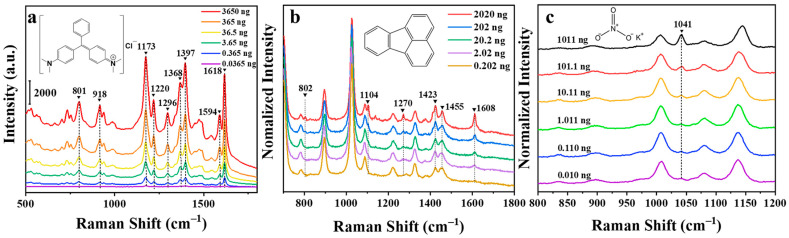
SERS spectra of malachite green (**a**), fluoranthene (**b**), and potassium nitrate (**c**) on silver nanowire membranes treated by 15 CV cycles at 20 °C.

Malachite green, both a dye and a fungicide, is a synthetic poisonous triphenylmethane chemical substance that is widely used in the textile and aquaculture industry. Since triphenylmethane substances have significant carcinogenic effects, they can enter the human body through bioaccumulation [51]. Malachite green is prone to being adsorbed on substrate material, the trace amount of malachite green was swabbed for SERS detection. Figure 7a is the SERS spectra of malachite green. The conventional Raman spectrum of malachite green is shown in Figure S11a for comparison. The inset shows the molecular structure of malachite green. The Raman characteristic peaks of malachite green are 801, 918, 1173, 1220, 1296, 1368, 1397, 1594, and 1618 cm^−1^ [52]. As is illustrated in Figure 7a, the characteristic peak intensity and the content of malachite green are positively correlated. The linear equation was exhibited in Figure S9d within the dynamic range of 0.0365–3.65 ng. Additionally, the calculated LOD of the malachite green mass was 0.00693 ng.

Polycyclic aromatic hydrocarbons (PAHs) are a common environmental and food organic pollutant with carcinogenic and teratogenic characteristics [53]. Such substances tend to accumulate in the human body and organisms and ultimately affect their health. In SERS detection, PAHs are rarely adsorbed near the surface of the SERS substrate. Therefore, it is difficult to detect PAHs using traditional methods. Mercaptans, common modifier materials, are relatively easy to form a molecular layer on the surface of precious metals, which can adsorb hydrophobic molecules such as polycyclic aromatic hydrocarbons in the vicinity of its surface in water and then achieve SERS detection [54]. Propyl mercaptan was selected as a surface modification molecule to enrich fluoranthene molecules during the swabbing extraction experiment. The trace amount of fluoranthene on the surface of a clean polyethylene packaging bag was collect by wiping and extracting for SERS detection. Figure 7b is the SERS spectra of fluoranthene on propyl mercaptan (normalized by Raman peak at 1025 cm^−1^ of propyl mercaptan). The conventional Raman spectrum of fluoranthene is displayed in Figure S11b for comparison. The characteristic peaks at 701 cm^−1^, 895 cm^−1^, 1025 cm^−1^, and others unmarked are attributed to propyl mercaptan [54]. The inset shows the molecular structure of fluoranthene. The characteristic peaks of fluoranthene are 802, 1104, 1270, and 1608 cm^−1^. Among them, 802 cm^−1^ is the C–H stretching vibration peak; 1104 and 1270 cm^−1^ are the C–H in-plane stretching vibration; 1608 cm^−1^ is the C–C stretching vibration peak [54]. The characteristic peak intensity of fluoranthene is proportional to its mass. As is displayed in Figure S9e, the linear equation was given within the dynamic range from 0.0202 to 2.02 ng. In addition, the LOD of the fluoranthene mass turned out 0.0810 ng.

Nitrate, a common oxidant in explosives, can be prone to explosion when it comes in contact with most organic substances. The presence of nitrate is a potential threat to public safety. The detection of nitrate can be equivalent to the detection of nitrate ion. As nitrate ions cannot be directly adsorbed on the surface of the substrate, the substrate modified with DDTC was used again to accumulate nitrate ion in swabbing extraction [12]. In this experiment, the trace amount of potassium nitrate on the clean aluminum sheet was collect by wiping and extracting for SERS detection. Figure 7c is the SERS spectra of potassium nitrate (normalized by the Raman peak at 1271 cm^−1^ of DDTC). The conventional Raman spectrum of potassium nitrate is shown in Figure S11c for comparison. The characteristic peak intensity of nitrate is proportional to the content of it. As illustrated in Figure S9f, the linear equation was computed, and the dynamic range is from 0.0110 to 1.10 ng. Moreover, the LOD of the fluoranthene mass was calculated to be 0.0273 ng.

### 3.6. DDA Simulations

**Figure 8:**
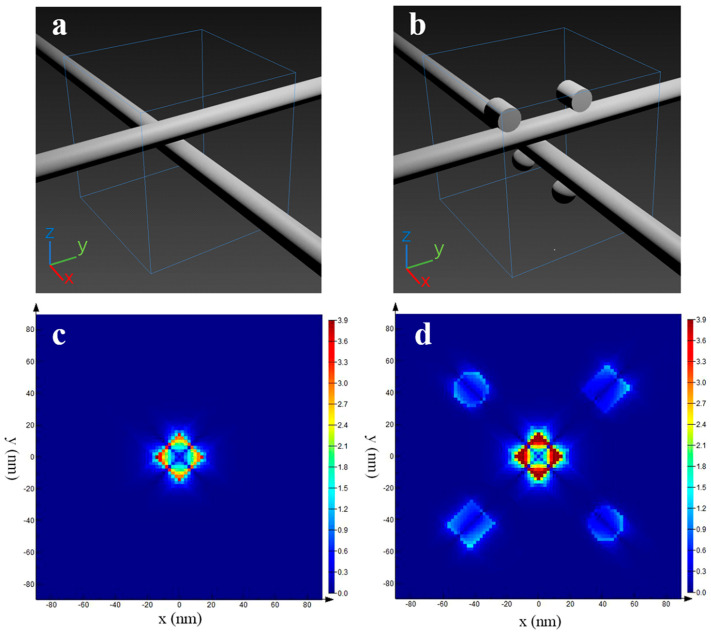
Three-dimensional (3D) images of theoretical models of silver nanowire membrane (**a**) and CV-treated silver nanowire membrane (**b**). DDA simulations of the electric field on untreated (**c**) and treated (**d**) silver nanowire membrane.

In order to further confirm that the electromagnetic enhancement effect of the CV-treated silver nanowire membrane is stronger than that of the untreated, theoretical simulation was carried under these two situations. In essence, the silver nanowire film is a stack of a large number of silver nanowires. After the CV treatment, some silver nanowires on the membrane surface may dissolve and crystallize into silver nanoparticles or silver nanorods on other silver nanowires surfaces. Based on this, the following models was established, which are shown in Figure 8a,b. Figure 8c,d shows the DDA simulations of the electric field on untreated (c) and treated (d) silver nanowire membranes. These simulated images indicate that not only the number of hot spots increases but also the electric field enhancement factor of the hot spots get higher in the CV-treated silver nanowire membrane. Therefore, the enhancement capability of the silver nanowires membrane is greatly improved after CV treatment, which is in agreement with the experimental results.

## 4. Conclusions

In this study, a highly sensitive flexible SERS substrate was developed by electrochemically treating a silver nanowire membrane. The process is both simple and time-saving. After CV treatment, silver nanoparticles and nanorods were formed on silver nanowires on the surface of the membrane, creating a high density of hot spots. The analytical enhancement factor of the substrate reached 1.24 × 10^9^ on average, which was 14.4 times higher than that of the untreated one. The substrate showed good stability when exposed in air for a period of time and continuously irradiated with a laser. PATP, crystal violet, thiram and sodium perchlorate were detected on this substrate with concentrations as low as 10^−11^ M, 10^−10^ M, 10^−10^ M, and 10^−8^ M in solution, respectively. The SERS substrate also can be used to swab analytes from the solid surface, and the detection limits are 0.0365, 0.2, and 0.01 ng for malachite green, fluoranthene, and potassium nitrate, respectively. The LODs of PATP, crystal violet, thiram, sodium perchlorate, malachite green, fluoranthene, and potassium nitrate were calculated to be 3.7 × 10^−12^ M, 5.1 × 10^−11^ M, 5.4 × 10^−11^ M, 6.3 × 10^−9^ M, 0.00693 ng, 0.0810 ng, and 0.0273 ng, respectively. In addition, the detection on real sample of crystal violet was proven to be feasible. This versatile SERS substrate could find more applications in the on-site inspection.

## Data Availability

Data sharing not applicable.

## Supplementary Materials

The following are available online at https://www.mdpi.com/2079-4991/11/3/672/s1, Figure S1: TEM images of silver nanowires at different magnifications. Figure S2: UV-Vis absorption spectrum of silver nanowires in water. Figure S3: Energy-dispersive X-ray spectrometry (EDS) mapping of silver nanowire membrane treated by CV with 15 cycles at 20 °C. SEM of the CV-treated silver nanowire membrane (a). The element distribution of Ag (green) and Cl (red) in this area (b,c). EDS spectrum of silver nanowire membrane treated by CV with 15 cycles at 20 °C (d). Figure S4: The current–potential curve (a), potential–time curve (b), current–time curve (c), and charge–time curve (d) of the silver nanowire membrane during electrochemical treatment. Figure S5: SEM images of silver nanowire membranes treated electrochemically at different temperatures: 16 °C (a), 18 °C (b), 20 °C (c), 22 °C (d), 24 °C (e), and 26 °C (f). Figure S6: SERS spectra of raw substrate (red) and substrate washed by sodium borohydride (blue). The black line is background. Table S1: The AEF of substrates prepared at different temperatures with the probe molecule of PATP. Figure S7: SERS intensities of PATP on the treated substrate at 1077 cm^−1^ form sampling in five locations. Figure S8: SERS intensity mapping of PATP on the treated substrate at 1077 cm^−1^ (right) and optical microscope image of the sampling area (left). Figure S9: The calibration curve of the detection of crystal violet (a), tetramethylthiuram disulfide (b), perchlorate (c), malachite green (d), fluoranthene (e), and nitrate (f). Figure S10: The conventional Raman spectrum of crystal violet and PATP. Figure S11: The conventional Raman spectra of malachite green (a), fluoranthene (b), and potassium nitrate (c). Figure S12: SERS spectra of treated (cycle number: 15; temperature: 20 °C) and untreated substrate when detecting crystal violet (a), thiram (b), sodium perchlorate (c), malachite green (d), fluoranthene (e), and potassium nitrate (f). Table S1: The AEF of substrates prepared in 15 cycles at different temperatures with the probe molecule of PATP. Table S2: The AEF of substrates prepared in different CV cycles at 20 °C with the probe molecule of PATP. Table S3: The AEF of substrates prepared in 15 cycles at 20 °C on detecting different analytes. Table S4: A comparison of performance of the developed SERS substrate with other sensors reported in the literature for the detection of crystal violet. Table S5: Determination of crystal violet in various spiked pond water samples using the developed SERS substrate.
