# Supplementary material for: Enhancing the Activity of Silver Nanowire Membranes by Electrochemical Cyclic Voltammetry as Highly Sensitive Flexible SERS Substrate for On-Site Analysis
Source: Nanomaterials (Basel). 2021 Mar 9;11(3):672. doi: 10.3390/nano11030672 (PMC7998130; doi:10.3390/nano11030672)
Supplement: Supplementary file 1 [file nanomaterials-11-00672-s001.pdf]

Supplementary Information

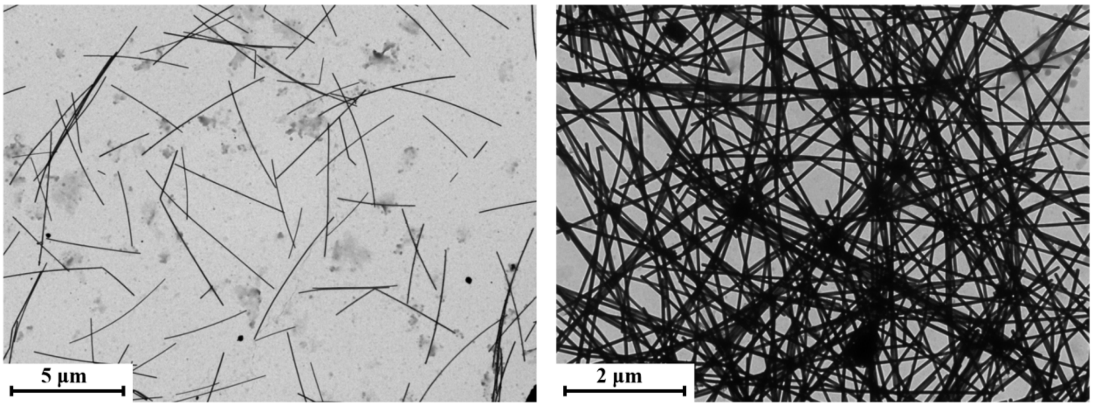

Figure S1. TEM images of silver nanowires at different magnifications.

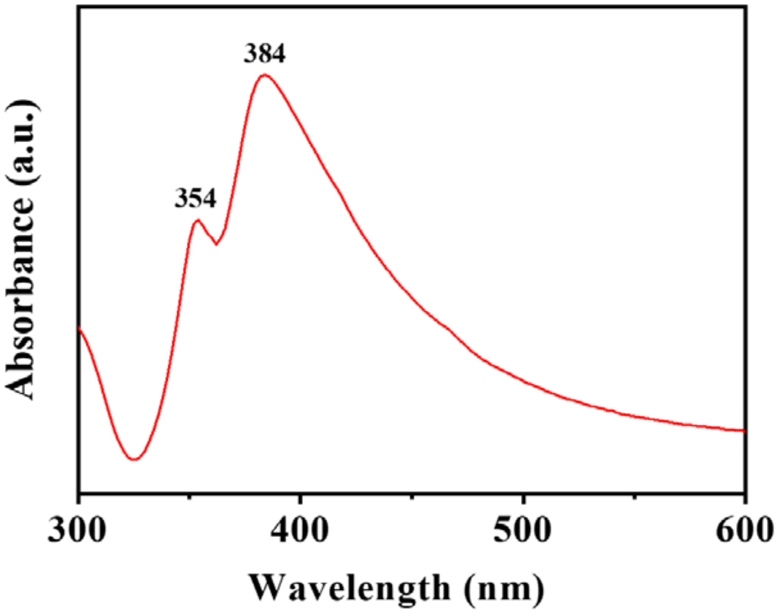

Figure S2. UV-Vis absorption spectrum of silver nanowires in water.

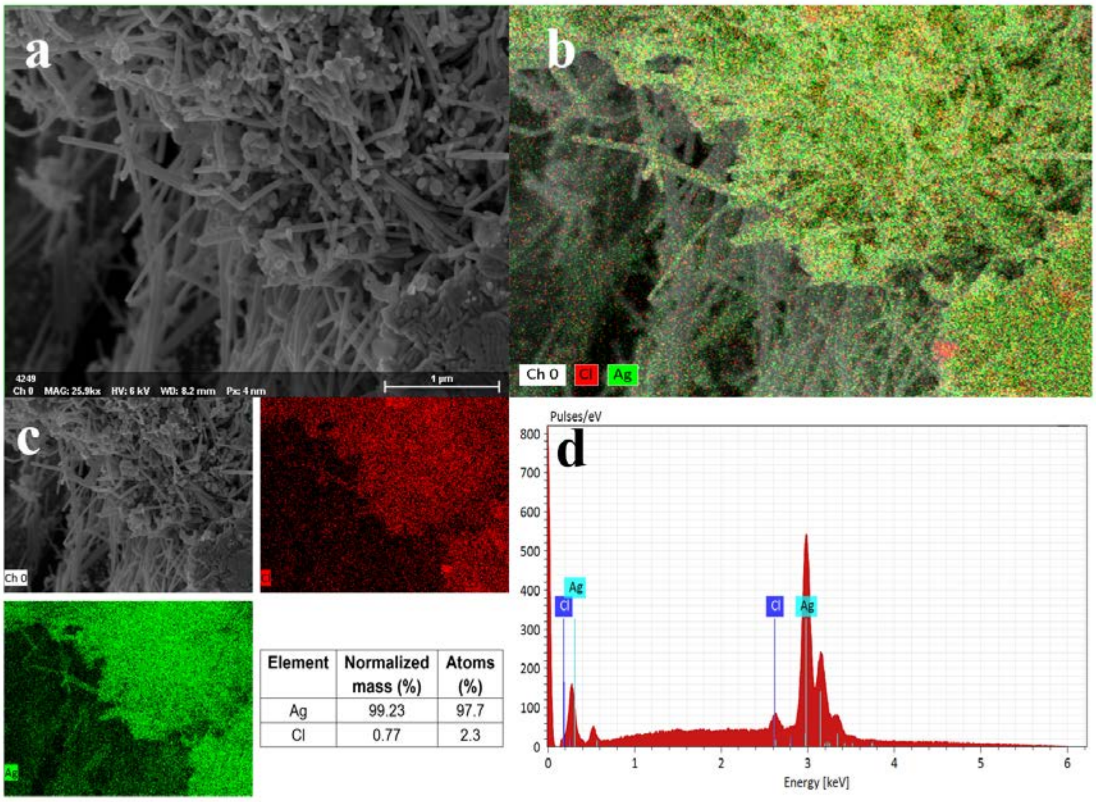

Element	Normalized mass (%)	Atoms (%)
Ag	99.23	97.7
Cl	0.77	2.3

Figure S3. EDS mapping of silver nanowire membrane treated by CV with 15 cycles at 20 °C. SEM of the CV treated silver nanowire membrane (**a**). The element distribution of Ag (green) and Cl (red) in this area (**b**, **c**). EDS spectrum of silver nanowire membrane treated by CV with 15 cycles at 20 °C (**d**).

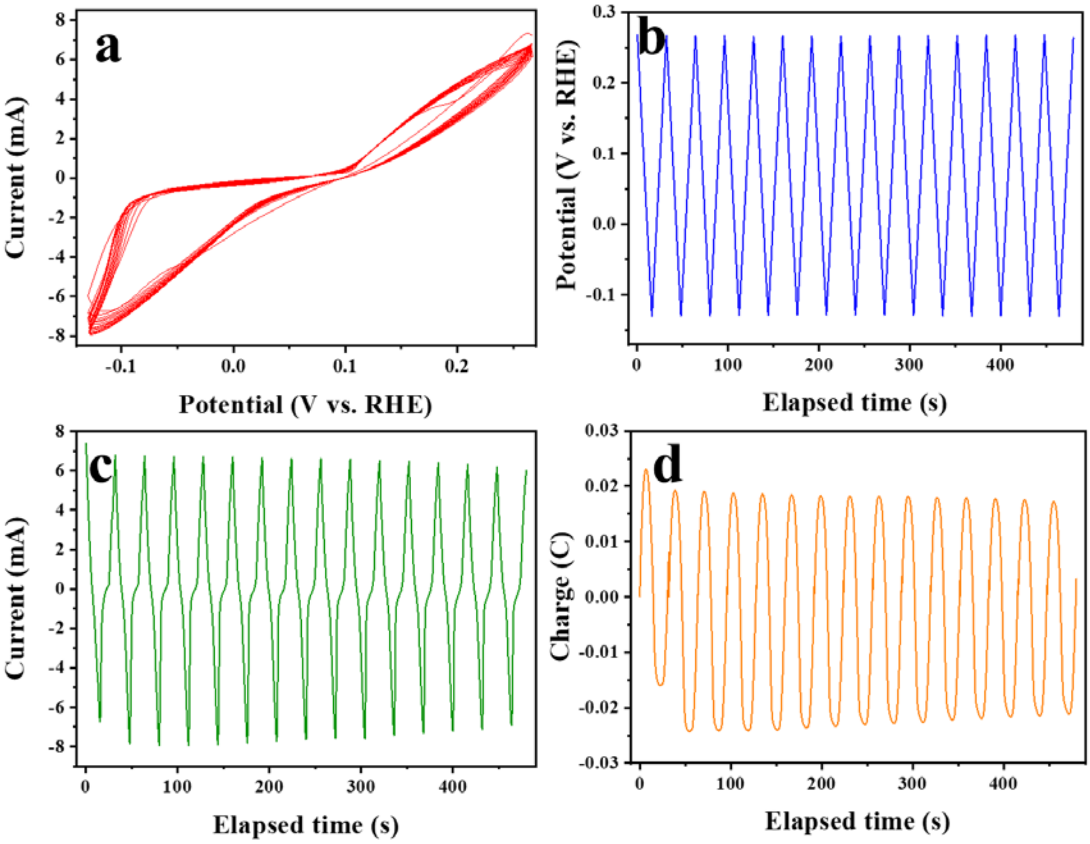

Figure S4. The current–potential curve (**a**), potential–time curve (**b**), current–time curve (**c**), and charge–time curve (**d**) of the silver nanowire membrane during electrochemical treatment.

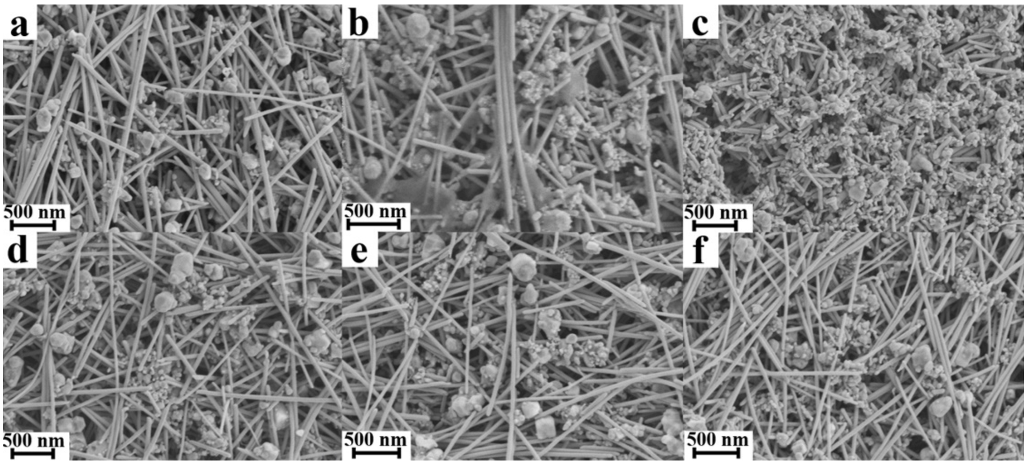

Figure S5. SEM images of silver nanowire membranes treated electrochemically at different temperatures: 16 °C (**a**), 18 °C (**b**), 20 °C (**c**), 22 °C (**d**), 24 °C (**e**), and 26 °C (**f**).

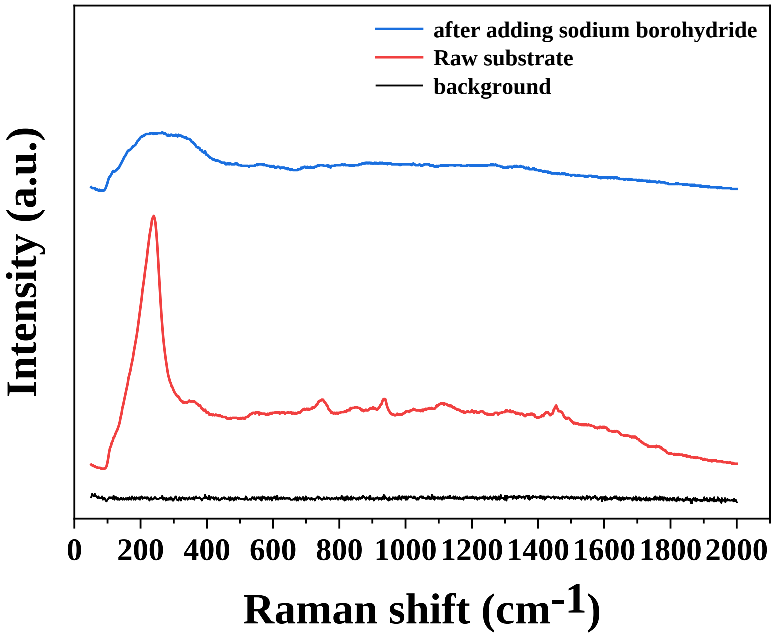

Figure S6. SERS spectra of raw substrate (red) and substrate washed by sodium borohydride (blue). The black line is background.

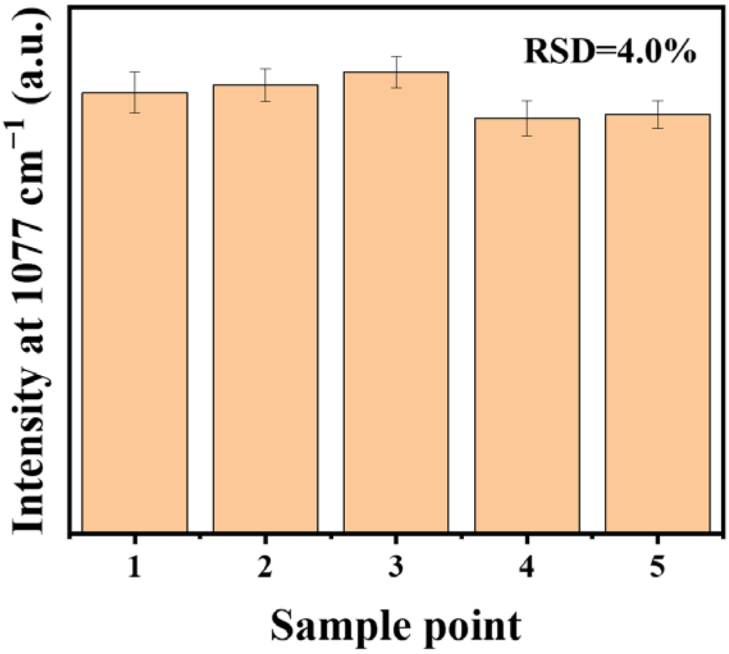

Figure S7. SERS intensities of PATP on the treated substrate at 1077 cm^{-1} form sampling in five locations.

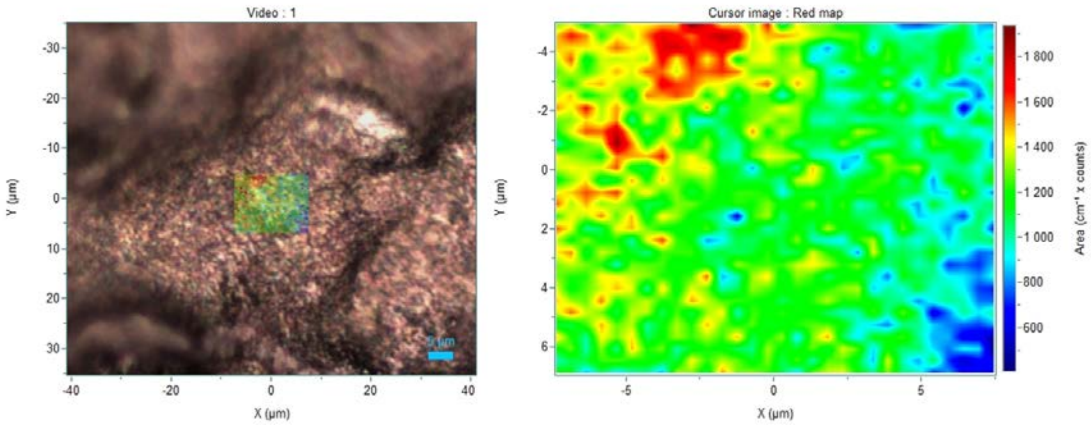

Figure S8. SERS intensity mapping of PATP on the treated substrate at 1077 cm^{-1} (**right**) and optical microscope image of the sampling area (**left**).

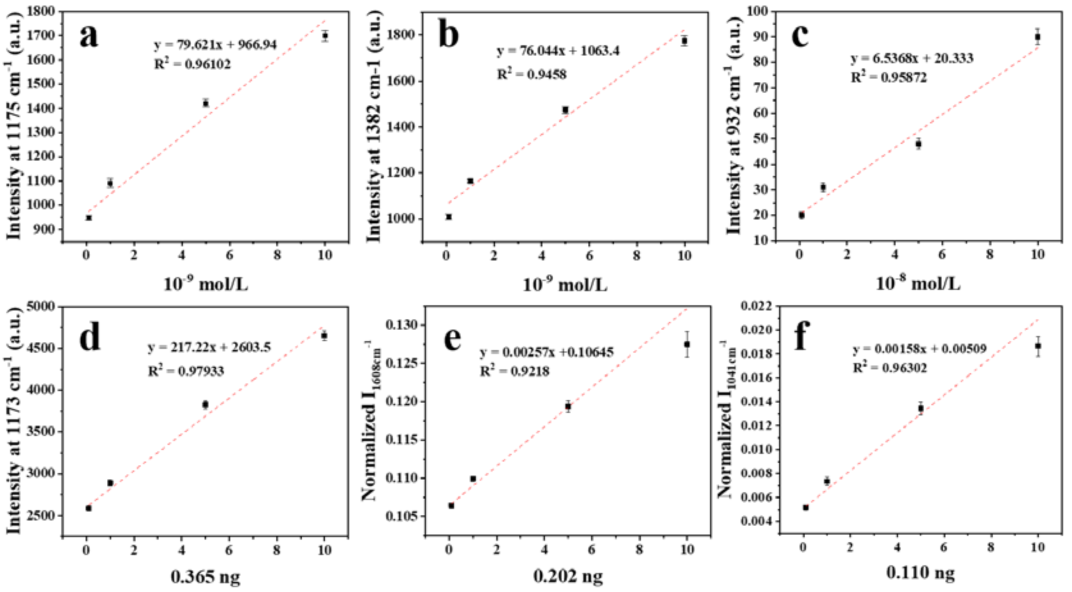

Figure S9. The calibration curve of the detection of crystal violet (**a**), tetramethylthiuram disulfide (**b**), perchlorate (**c**), malachite green (**d**), fluoranthene (**e**) and nitrate (**f**).

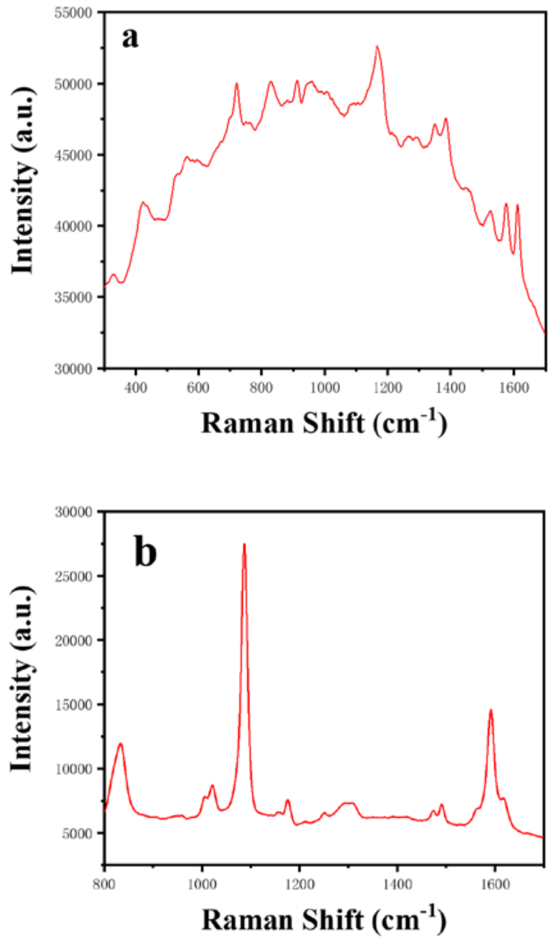

Figure S10. The conventional Raman spectra of crystal violet (**a**) and PATP (**b**).

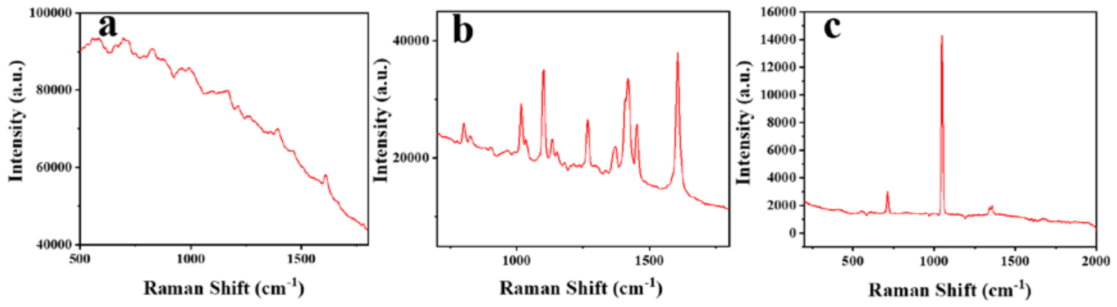

Figure S11. The conventional Raman spectra of malachite green (**a**), fluoranthene (**b**) and potassium nitrate (**c**).

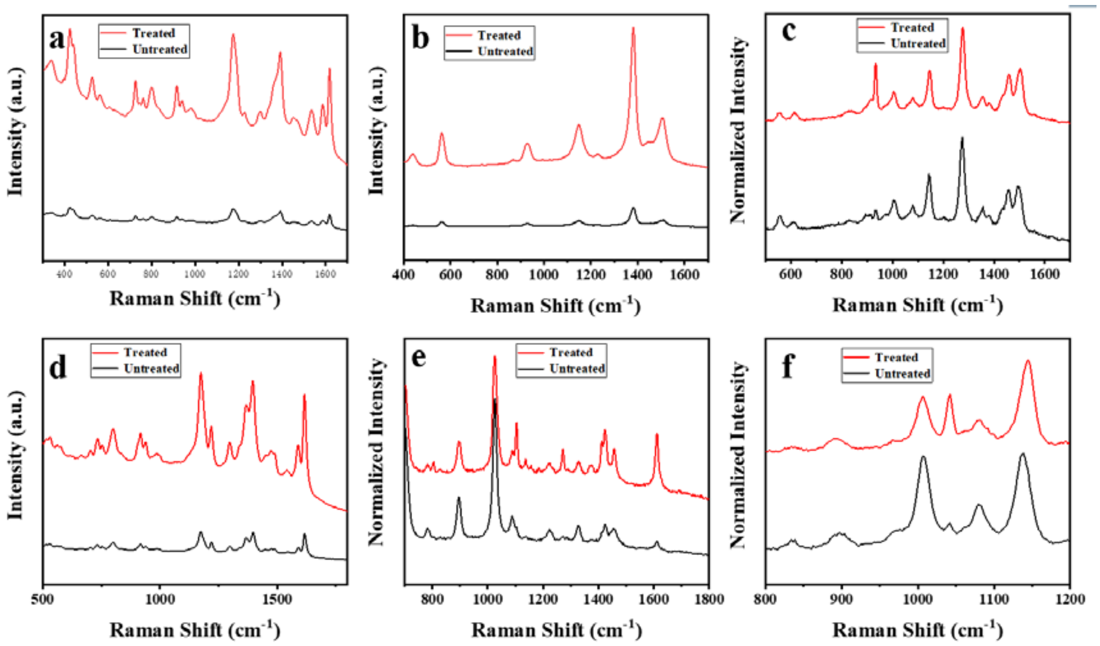

Figure S12. SERS spectra oftreated (cycle number: 15; temperature: 20 °C) and untreated substrate when detecting crystal violet (**a**), thiram (**b**), sodium perchlorate (**c**), malachite green (**d**), fluoranthene (**e**) and potassium nitrate (**f**).

Table S1. The AEF of substrates prepared in 15 cycles at different temperatures with the probe molecule of PATP.

Temperature	untreated	16 °C	18 °C	20 °C	22 °C	24 °C	26 °C
AEF	8.60×10^7	4.29×10^8	5.66×10^8	1.24×10^9	7.31×10^8	4.00×10^8	2.53×10^8
Magnification	1.00	4.99	6.59	14.4	8.50	4.65	2.94

Table S2. The AEF of substrates prepared in different CV cycles at 20 °Cwith the probe molecule of PATP.

Temperature	untreated	5 cycles	10 cycles	15 cycles	20 cycles	25 cycles
AEF	8.60×10^7	4.92×10^8	8.33×10^8	1.21×10^9	9.09×10^8	6.56×10^8
Magnification	1.00	5.72	9.69	14.1	10.6	7.63

Table S3. The AEF of substrates prepared in 15 cycles at 20 °C on detecting different analytes.

Analyte	crystal violet	thiram	sodium perchlorate	malachite green	fluoranthene	potassium nitrate
AEF	1.16×10^9	1.07×10^9	9.42×10^8	1.02×10^9	8.76×10^8	9.68×10^8

Table S4. A comparison of performance of the developed SERS substrate with other sensors reported in literature for the detection of crystal violet.

Method	LOD	Linear range	Ref.
UPLC with EIT-MS	0.15 μg/kg	0.5 to 10 μg/kg	[1]
CuS film & $K_2S_2O_8$	3.2×10^{-9} mol/L	5.0×10^{-8} to 1.0×10^{-5} mol/L	[2]
Spectrophotometric method	0.01312 μg/L	0.75–10.00 μg mL^{-1}	[3]
N-GQDs and $K_2S_2O_8$	45 nmol/L	0.05–5 μmol/L	[4]
Cyclic voltammetry treated silver nanowire membrane	5.1×10^{-11} mol/L	10^{-10} to 10^{-8} mol/L	Current work

Table S5. Determination of crystal violet in various spiked pond water samples using the developed SERS substrate.

Spiked levels (10^{-9} mol/L)	Found ± SD (10^{-9} mol/L)	Recovery (%)	RSD (%)
0.5	0.4403 ± 0.0264	88.07	5.99
1	0.9804 ± 0.0427	98.04	4.36
5	5.0748 ± 0.0854	101.5	1.68
10	8.9180 ± 0.1155	89.18	1.30

*SD, standard deviation; RSD, relative standard deviation

References:

[1] Zhang, Z.G.; Shi, B.; Chen, L.P.; Lin, L.Y.; Zhou, Y. Simultaneous determination and confirmation of malachite green, crystal violet and their leuco metabolites residues in aquatic product by liquid chromatography-visible detection and tandem mass spectrometric detection. *Chin. J. Anal. Chem.* **2006**, *34*, 663–667.

[2] Lan, Q.F.; Li, Q.Y.; Zhang, X.Y.; Chen, Z.D. A novel electrochemiluminescence system of CuS film and $K_2S_2O_8$ for determination of crystal violet. *J. Electroanal. Chem.* **2018**, *810*, 216–221.

[3] Sar, S.K.; Verma, C.; Pandey, P.K.; Bhui, A. Reliable Technique for the Determination of Sodium Dodecyl Sulphate by Crystal Violet in Relation to the Effluents of Durg-Bhilai Region. *J. Chin. Chem. Soc. (Weinheim, Ger.)* **2009**, *56*, 1250–1256.

[4] Chen, X.H.; Shan, X.L.; Lan, Q.F.; Chen, Z.D. Electrochemiluminescence Quenching Sensor of a Carboxylic Carbon Nanotubes Modified Glassy Carbon Electrode for Detecting Crystal Violet Based on Nitrogen-doped Graphene Quantum Dots@Peroxydisulfate System. *Anal. Sci.* **2019**, *35*, 929–934.
